# The impact of nursing-led emotional preparation on mental health after total hip arthroplasty

**DOI:** 10.3389/fpsyg.2025.1693111

**Published:** 2025-11-05

**Authors:** Elisabet Ripoll-Romero, Zaida Agüera, Montserrat Puig-Llobet, Jordi Galimany-Masclans

**Affiliations:** ^1^Department of Orthopedic Surgery, Hospital Clinic de Barcelona, Barcelona, Spain; ^2^Departament d’Infermeria de Salut Pública, Salut Mental i Maternoinfantil, Facultat d’Infermeria, Universitat de Barcelona, Barcelona, Spain; ^3^Research Group in Mental Health, Psychosocial and Complex Nursing Care (NURSEARCH), Facultat d’Infermeria, Universitat de Barcelona, Barcelona., Spain; ^4^Psychoneurobiology of Eating and Addictive Behaviors Group, Neurosciences Programme, Bellvitge Biomedical Research Institute (IDIBELL), Barcelona, Spain; ^5^CIBER Fisiopatología Obesidad y Nutrición (CIBERobn), Instituto de Salud Carlos III, Madrid, Spain

**Keywords:** total hip arthroplasty, anxiety, nursing intervention, postoperative recovery, quality of life

## Abstract

**Background:**

Postoperative anxiety and depression are common following total hip arthroplasty (THA) and can negatively affect recovery. While pharmacological management is standard, non-pharmacological interventions may offer additional benefits without adverse side effects.

**Objective:**

To assess the effectiveness of a nurse-led video-based intervention on anxiety and depression symptoms and perceived quality of life in patients undergoing THA, compared to standard care.

**Methods:**

A quasi-randomized controlled trial was conducted with 131 participants undergoing elective THA, randomly assigned to an intervention group (IG; *n* = 67) receiving a preoperative nursing intervention focused on emotional preparation and information, and a control group (CG; *n* = 64) receiving usual care. Psychopathological symptoms were assessed using the Hospital Anxiety and Depression Scale (HADS), and quality of life was measured using the EQ-5D-5L. Assessments occurred at baseline (pre-surgery), post-intervention (hospital discharge), and one-month follow-up. General Linear Model (GLM) analyses were used for within- and between-group comparisons.

**Results:**

No significant differences in anxiety or depression symptoms were found between baseline and hospital discharge in either group. Both groups showed significant improvement in HADS scores and all EQ-5D-5L dimensions at one-month follow-up. Although the IG initially appeared to show greater improvement in depression symptomatology and in the ‘usual activities’ dimension compared to the CG, these differences were no longer statistically significant after adjusting for baseline depression. No other significant between-group differences were observed.

**Conclusion:**

The nurse-led video-based intervention did not produce immediate emotional benefits but was associated with improved functional recovery at 1 month; however, it has not been shown to be more effective than usual care. These findings suggest that targeted nursing interventions may support postoperative recovery, particularly in functional outcomes, while emotional effects remain inconclusive and warrant further investigation. Importantly, the video format offers a more sustainable and cost-effective approach compared to printed materials, reducing the need for physical handouts while maintaining structured patient education.

## 1 Introduction

Osteoarthrosis (OA) is a chronic and degenerative disease characterized by wear and tear of the joints. It is a major cause of pain, disability, and decreased quality of life (Vidal Fuentes, 2021). Total hip arthroplasty (THA) and total knee arthroplasty (TKA) represent effective alternatives for the treatment and pain control of patients with osteoarthritis (Impieri et al., 2025). The fast-track program is an enhanced recovery intervention that consists of the active participation of patients in their recovery and immediate post-surgical mobilization. This fast-track recovery is achieved using preoperative education through an educational workshop, the type of analgesia used, and the empowerment of the patient in their recovery (Matute Crespo and Montero Matamala, 2021).

Discharge from hospital 24 h after arthroplasty surgery has been shown to have many benefits, such as faster rehabilitation, improved patient satisfaction, reduced reliance on hospital resources, and potential for cost reductions (Scully et al., 2020). However, despite the benefits associated with earlier recovery, it has also been associated with elevated levels of pre- and postoperative anxiety (Arshi et al., 2022; Cronin et al., 2022). Anxiety is a common emotional response to perceived threats, involving both cognitive, emotional, behavioral, and physiological changes. Surgery is a significant stressor, and patients often experience anxiety both before and after the THA (Öztekin et al., 2025). Previous studies have shown that targeted psychosocial interventions, including preoperative information and emotional support, can reduce anxiety and improve postoperative outcomes (Deng et al., 2022; Oberai et al., 2021; Yang et al., 2025; Sato et al., 2024).

However, most research has focused on preoperative anxiety, with limited evidence on the effects of targeted interventions during the early postoperative period and after discharge. The few studies addressing psychosocial interventions facing emotional wellbeing at post-operative phases (Sinatti et al., 2022; Zhang et al., 2022; Zhong et al., 2021) showed that adequate preoperative information reduces both preoperative and postoperative anxiety (Giardina et al., 2020).

Several studies have shown that videos designed to emotionally prepare patients or guide them in their recovery process can reduce post-surgical anxiety, promote understanding of treatment and improve adherence to medical recommendations (Wasim et al., 2024; Kim et al., 2021). However, few works have focused on its application in the immediate postoperative period and even fewer have been led and applied by nursing staff, which reinforces the originality and relevance of this study.

Despite the recognized importance of nursing interventions in managing preoperative anxiety, there remains a significant gap in evidence regarding effective strategies to reduce anxiety in the immediate postoperative period, particularly just before hospital discharge. Given that early discharge after THA is increasingly associated with heightened postoperative anxiety, it is crucial to explore interventions that support patients during this vulnerable phase. Furthermore, understanding how these interventions impact patients’ perceived quality of life in the short term can provide valuable insights for improving postoperative care. Therefore, this study aimed to assess the short-term effectiveness of a targeted nursing intervention in reducing anxiety symptoms during the surgical process in patients undergoing THA, as well as to assess the perceived quality of life of these patients in comparison with those receiving the treatment as usual.

We hypothesized that providing a brief targeted nursing intervention will be more effective than usual care in reducing anxiety symptoms during the surgical process, but also in improving their recovery and patients’ perceived quality of life.

## 2 Methods

### 2.1 Design

A two-arms quasi-randomized design was conducted. Ethical and institutional approvals were received from Ethical Committee at the Hospital Clínic of Barcelona (ref HCB/2023/0015). All participants signed the informed consent prior to participating in the study. This RCT was registered in the ClinicalTrials.gov Protocol Registration and Results System (PRS) (NCT05882227). The RCT adhered to the Consolidated Standards of Reporting Trials (CONSORT) 2010 guidelines for reporting randomized controlled trials.

### 2.2 Setting and sample

The sample consists of all patient’s consecutive admitted for primary THA surgery at the Orthopedics Surgery and Traumatology Unit of a tertiary hospital between October 1, 2023, and September 31, 2024. The inclusion criteria were: (1) patients scheduled with signed consent for primary THA intervention, (2) age over 18 years, (3) willingness to participate in the study, and (4) completion of all questionnaires without missing data. The exclusion criteria were: (1) patients who were discharged to a nursing home, (2) patients with cognitive or mental disorders, and (3) patients unable to read or write, which would hinder the fulfillment of the psychometric assessment.

**Figure 1:**
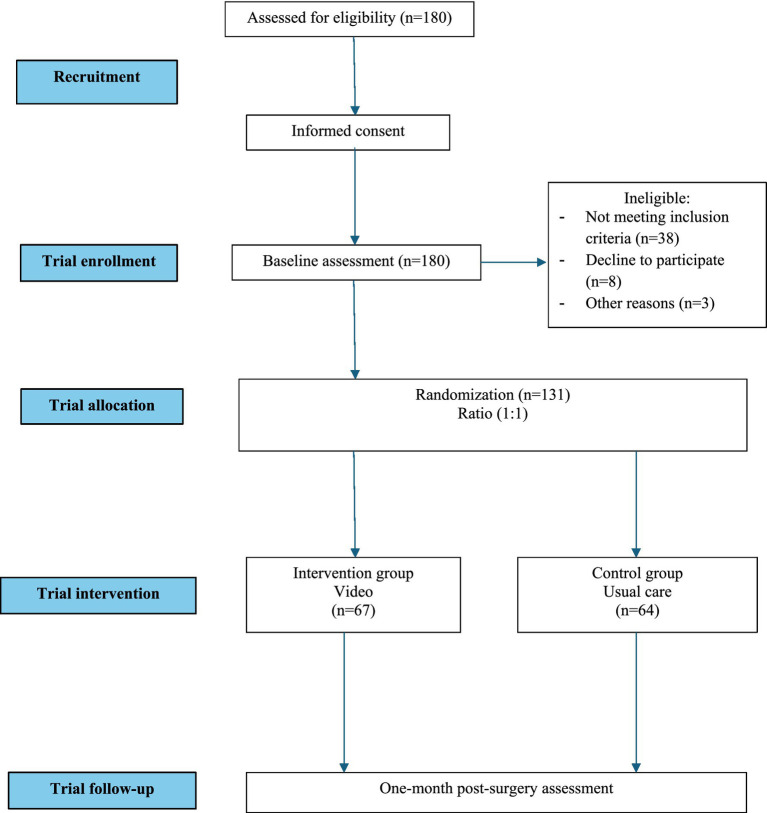
Flow diagram of patient selection.

No formal sample size or power calculation was performed, as this study was exploratory, and recruitment was limited by the available patient population during the study period. Patients admitted to the unit during even weeks will be assigned to the intervention group (IG) and patients admitted during odd weeks will be assigned to the control group (CG). Blinding was not performed because the intervention involved the nurse showing a video to the IG, while the CG received usual care (a leaflet with nursing recommendations at discharge). To reduce potential bias and facilitate hospital logistics, the intervention was assigned on alternating weeks (even vs. odd weeks). A total of 180 patients were enrolled in the study, of whom 131 completed the intervention (Figure 1).

### 2.3 Instruments

#### 2.3.1 Demographic variables

Demographic variables included gender, age, cohabitation status, academic level, history of psychiatric problems, and use of psychopharmacological treatment.

#### 2.3.2 Hospital anxiety and depression scale (HADS)

The Hospital Anxiety and Depression Scale (HADS) is a self-administered instrument created to identify symptoms of anxiety and depression in patients without diagnosed psychiatric pathology (Herrero et al., 2003). It consists of 2 subscales: Depression (HADS-D) and Anxiety (HADS-A), each with seven items. The score of each subscale can vary between 0 and 21, since each item presents four response options, ranging from absence/minimum presence = 0 to maximum presence = 3. The higher the score obtained, the greater the intensity or severity of the symptoms. The internal consistency (coefficient alpha) for the current sample was 0.80 for HADS-A and 0.83 for HADS-D subscale.

#### 2.3.3 The 5-level EQ-5D version (EQ-5D-5L)

This questionnaire is an instrument for measuring health-related quality of life (EuroQol, 2024). This questionnaire can be used both in relatively healthy individuals (general population) and in groups of patients with different pathologies. The individual assesses his or her own state of health, first in terms of severity level by dimensions and then on a more general visual analog scale (VAS). A third element is the index of social values obtained for each state of health generated by the instrument. The descriptive system contains five health dimensions (mobility, self-care, activities of daily living, pain/discomfort, and anxiety/depression). The internal consistency of our sample was 0.78.

### 2.4 Procedure and data collection

When patients who met the inclusion criteria arrived at the orthopedic ward, they were provided with both written and oral information about the study. Each patient was informed that participation was voluntary and that they could withdraw their consent at any time without giving a reason. The information was provided by a registered nurse on the ward who is a member of the study’s research team.

When patients agreed to participate in the study, they were assessed at baseline with the psychometric battery, both in IG and in CG, during the visit with the advanced practice nurse in outpatient clinics. To ensure randomization, patients who underwent surgery in even weeks received the additional nursing intervention proposed in this study (IG), while those who had surgery in odd weeks received treatment as usual (CG). Participants completed outcomes questionaries face-to-face preoperatively before hospital discharge post-measure. The time elapsed between the first and the second data collection was 1 month. Finally, coinciding with the one-month follow-up after the surgery, the participants were assessed again with the same battery of questionnaires, both CG and IG.

### 2.5 Intervention modalities

The usual post-surgery intervention consisted of providing a document titled “Optimized hip prosthesis recovery program; information for patients, relatives or caregivers.” This document consists of a leaflet where the patient can consult general health recommendations, care of the surgical wound, how to mobilize, warning signs and information on drug treatment. Instead, the IG received the brief nursing intervention that involved watching a video based on the content of the same document. The video, which lasted 5 min and 9 s, was made available on the in-room tablets used for television in the hospital ward. Patients began watching the video upon arrival to the unit and were able to view it as many times as they wished throughout their hospital stay. The intervention was reinforced at the time of discharge, coinciding with the nurse’s routine delivery of post-operative recommendations. The video was divided into two parts: the first part explained what happens in the next hours in the unit, the documentation to be delivered at discharge and the pharmacological treatment. The second part focused on wound care after discharge, discharge recommendations, symptoms that may appear after the surgery, and alarm signed. This procedure was carried out by the nursing team, composed of two registered nurses and one nursing care technician.

### 2.6 Statistical analyses

Statistical analyses were carried out with SPSS v.30 for Windows. First, we conducted a Kolmogorov–Smirnov normality test to check if there was a normal distribution and to determine which statistic to use. Sociodemographic and clinical characteristics of the sample were analyzed by means of frequencies and percentages and means and standard deviation (SD) for categorical and quantitative variables, respectively. Baseline differences between the two groups (IG vs. CG) were analyzed with Chi-square for categorical variables and Student’s *t*-test for continuous variables.

To assess intervention effects over time, we first used 2×3 repeated-measures ANOVA (RM-ANOVA) with group (IG vs. CG) as the between-subject factor and time (baseline, hospital discharge, one-month follow-up) as the within-subject factor. Mauchly’s test of sphericity was applied for each outcome, and Greenhouse–Geisser corrections were used when sphericity was violated. Group×Time interactions were reported for each outcome variable. Statistical significance was set at *p* < 0.05. Effect sizes was estimated using Cohen’s *d* continuous variables, considering a moderate effect for |*d*| > 0.50 and a large effect for |*d*| > 0.80. For repeated measures, eta squared (η^2^) was reported as a measure of effect size, with values around 0.01 considered small, around 0.06 moderate, and above 0.14 large, according to commonly used benchmarks.

Subsequently, sensitivity analyses were conducted using univariate ANCOVA models to adjust for potential confounders, specifically baseline depression scores and week of inclusion. Estimated marginal means were reported for each group, and between-group comparisons were adjusted using the Bonferroni-adjusted. These analyses allowed evaluation of the intervention effect while controlling for baseline depressive symptoms and temporal variability, providing a more accurate assessment of functional and emotional outcomes. Statistical significance was set at *p* < 0.05. Effect sizes was estimated using Cohen’s *d* continuous variables, considering a moderate effect for |*d*| > 0.50 and a large effect for |*d*| > 0.80. For repeated measures, eta squared (η^2^) was reported as a measure of effect size, with values around 0.01 considered small, around 0.06 moderate, and above 0.14 large, according to commonly used benchmarks.

## 3 Results

### 3.1 Demographic and clinical characteristics

No significant differences were found between the CG and the IG in mean age neither other sociodemographic variable at baseline. A summary of participant demographic and clinical characteristics is provided in Table 1.

**Table 1:** Sociodemographic and clinical characteristics of the sample (*n* = 131).

Variable	Total		CG		IG		*χ* ^2^	*p*-value
	*n* = 131		*n* = 64		*n* = 67			
	*n*	%	*n*	%	*n*	%		
Gender								
Male	76	58	42	65.6	34	50	2.975	0.085
Female	55	42	22	34.4	33	50		
Cohabitation								
Alone	24	18.3	10	15.6	14	21.2	1.085	0.781
1st grade	97	74	50	78.2	48	71.2		
Others	10	7.6	4	6.3	5	7.5		
Studies								
Primary	34	26	13	20.3	21	30.3	2.771	0.428
High school	49	37.4	25	39.1	24	36.4		
College	48	36.6	26	40.6	22	33.3		
Psychiatric problems								
Yes (anxiety, depression, others)	21	16	10	15.7	11	16.3	5.074	0.280
No	110	84	54	84.4	56	83.3		
Sleep problems								
Yes	6	4.6	1	1.6	5	7.6	2.607	0.106
No	125	95.4	63	98.4	62	92.4		
Psychopharmacological treatment								
Yes	23	17.6	9	14.1	14	21.2	0.974	0.324
No	108	82.4	55	86	53	78.8		

	Mean	SD	Mean	SD	Mean	SD	F	*p*-value
Age	64.98	11.53	63.08	12.73	66.81	10.02	2.793	0.064

SD, standard deviation; CG, control group; IG, intervention group. ^*^Significance *p* < 0.05.

### 3.2 Presurgical psychopathology and quality of life

Table 2 presents the baseline characteristics of the CG and IG across the HADS and EQ-5D-5L. The results showed no statistically significant differences between groups at baseline in anxiety, as measured by HADS. However, the CG exhibited higher depressive symptoms on the HADS depression subscale compared to the IG. Regarding the EQ-5D-5L dimensions, no significant differences were found between groups in mobility, self-care, usual activities, or pain/discomfort. Notably, the anxiety/depression dimension of this scale indicated a significant difference, with the IG reporting fewer problems than the CG. However, the effect size (measured by Cohen’s *d*) for both significant differences was very small.

**Table 2:** Comparison of baseline scores on HADS and EQ-5D-5L.

	CG (*n* = 64)	IG (*n* = 67)	t-Student (*t*)	*p*-value	*/d/*
	Mean (SD)	Mean (SD)			
HADS					
HADS-A	7.42 (4.12)	6.27 (4.09)	1.608	0.11	0.28
HADS-D	6.84 (3.73)	5.21(4.29)	2.324	**0.022**	0.41
EQ-5D-5L					
Mobility	3.44 (0.833)	3.25 (0.927)	1.192	0.236	0.20
Self-care	2.69 (1.02)	2.58 (1.01)	0.592	0.555	0.10
Usual activities	3.47 (0.975)	3.36 (1.05)	0.622	0.535	0.10
pain/discomfort	3.64 (0.843)	3.73 (0.750)	−0.651	0.517	−0.11
Anxiety/depression	2.61 (1.25)	2.12 (1.20)	2.28	**0.024**	0.39

SD, standard deviation; CG, control group; IG, intervention group; HADS, Hospital Anxiety and Depression Scale; HADS-A, Anxiety Scores; HADS-D, Depression Scores; EQ-5D-5L, EuroQol-5 dimensions-5 levels. *Bold values indicate statistically significant differences (*p* < 0.05). Cohen’s *d* considering a moderate effect for |*d*| > 0.50 and a large effect for |*d*| > 0.80.

### 3.3 Changes in psychopathological symptoms and quality of life after surgery in the IG and the CG

Table 3 presents the changes in psychopathology and quality of life over time in both the IG and the CG. Regarding psychopathology, assessed using the HADS, no statistically significant differences were observed between pre- and postoperative scores for either group (CG or IG) on either the HADS-A or HADS-D subscales. However, both groups showed statistically significant improvements from preoperative to one-month follow-up, as well as from postoperative to one-month follow-up, on both subscales.

**Table 3:** Within-subject comparison of measures at baseline, hospital discharge, and one-month follow-up in the IG and CG.

Variable	Baseline	Po	1- month follow-up	Post hoc comparison					
				Baseline vs. post-discharge		Baseline vs. 1- month follow-up		Post-discharge vs. 1- month follow-up	
	Mean (SD)	Mean (SD)	Mean (SD)	Mean (SD)	*p*-value	Mean (SD)	*p*-value	Mean (SD)	*p*-value
CG (*n* = 64)									
HADS									
HADS-A	7.42 (4.12)	7.58 (4.32)	4.23 (3.54)	−0.156 (3.78)	0.371	3.18 (4.08)	**<0.001**	3.34 (3.94)	**<0.001**
HADS-D	6.84 (3.72)	6.30 (4.46)	4.17 (3.54)	0.547 (3.01)	0.076	2.67 (4.15)	**<0.001**	2.12 (4.46)	**<0.001**
EQ-5D-5L									
Mobility	3.44 (0.83)	1.56 (0.64)	1.66 (0.70)	1.87 (1.01)	**<0.001**	1.78 (1.11)	**<0.001**	−0.094 (0.43)	0.083
Self-care	2.69 (1.02)	1.45 (0.59)	1.47 (0.62)	1.23 (1.17)	**<0.001**	1.21 (1.21)	**<0.001**	−0.016 (0.38)	0.742
Usual activities	3.47 (0.98)	1.83 (0.79)	1.92 (0.97)	1.64 (1.32)	**<0.001**	1.54(1.45)	**<0.001**	−0.094 (0.43)	0.083
pain/discomfort	3.64 (0.84)	1.78 (0.79)	1.81 (0.79)	1.85 (0.89)	**<0.001**	1.82 (0.89)	**<0.001**	−0.031 (0.39)	0.531
Anxiety/depression	2.61 (1.25)	1.47 (0.73)	1.44 (0.75)	1.14 (1.41)	**<0.001**	1.17 (1.37)	**<0.001**	0.031 (0.25)	0.321
IG (*n* = 67)									
HADS									
HADS-A	6.27 (4.09)	6.45 (3.78)	3.25 (3.22)	−0.179 (3.19)	0.319	3.016 (4.16)	**<0.001**	3.19 (3.35)	**<0.001**
HADS-D	5.21 (4.29)	5.48 (3.99)	2.99 (3.39)	−0.269 (3.40)	0.260	2.24 (3.85)	**<0.001**	2.49 (3.86)	**<0.001**
EQ-5D-5L									
Mobility	3.25 (0.93)	1.52 (0.73)	1.57 (0.72)	1.73 (1.32)	**<0.001**	1.68 (1.30)	**<0.001**	−0.045 (0.21)	0.083
Self-care	2.58 (1.01)	1.40 (0.65)	1.46 (0.79)	1.17 (1.15)	**<0.001**	1.11 (1.21)	**<0.001**	−0.069 (0.49)	0.321
Usual activities	3.36 (1.05)	1.61 (0.70)	1.52 (0.70)	1.74 (1.14)	**<0.001**	1.83 (1.14)	**<0.001**	0.090 (0.29)	**0.013**
pain/discomfort	3.73 (0.75)	1.73 (0.77)	1.69 (0.76)	2 (0.98)	**<0.001**	2.04 (0.98)	**<0.001**	0.045 (0.32)	0.260
Anxiety/depression	2.12 (1.20)	1.42 (0.78)	1.52 (0.93)	0.701 (1.32)	**<0.001**	0.597 (1.31)	**<0.001**	−0.104 (0.61)	0.163

SD, standard deviation; CG, control group; IG, intervention group; HADS, Hospital Anxiety and Depression Scale; HADS-A, Anxiety Scores; HADS-D, Depression Scores; EQ-5D-5L, EuroQol-5 dimensions-5 levels. *Bold values indicate statistically significant differences (*p* < 0.05).

Regarding quality of life, assessed using the EQ-5D-5L, both groups (CG and IG) showed statistically significant improvements in all subscales (i.e., mobility, self-care, usual activities, pain/discomfort, and anxiety/depression) at hospital discharge. These improvements remained stable during the one-month follow-up period. Notably, only the IG showed a further statistically significant improvement in the ‘usual activities’ subscale between hospital discharge and the one-month follow-up, indicating continued functional recovery in this domain.

### 3.4 Between-group comparisons (CG vs. IG) based on general linear model analysis

Between-group comparisons using General Linear Model analysis revealed no statistical differences in anxiety (HADS-A) but a statistically significant difference in depressive symptoms at one-month follow-up, with the CG reporting higher scores on the HADS-D subscale compared to the IG. The effect size for this difference was very small, suggesting limited clinical relevance. In addition, after adjusting for baseline depression and week of inclusion, no significant differences were observed between groups in HADS-D at discharge (EMMeans: CG = 5.72, IG = 6.03; difference IG–CG = 0.32, 95% CI − 1.40 to 0.77; *p* = 0.562) or at 1 month follow-up (EMMeans: CG = 3.90, IG = 3.25; difference = −0.65, 95% CI − 1.76 to 0.46; *p* = 0.252). Baseline scores were a significant predictor of post-intervention values (*p* < 0.001), whereas week of inclusion (even weeks for IG and odd weeks for CG) had no significant effect (post: *F* = 0.81, *p* = 0.369; 1 month: *F* = 1.31, *p* = 0.255). To ensure transparency and robustness, the adjusted analyses are reported in the Supplementary Table S1, while the Table 4 presents the original unadjusted comparisons for consistency.

**Table 4:** Between-group comparisons (CG vs. IG) based on general linear model analysis.

outcome variable	Group means (M ± SD)	F	*p*-value	η^2^
HADS-A	CG: 4.24 ± 3.54 IG: 3.25 ± 3.22	3.81	0.053	0.029
HADS-D	CG: 4.17 ± 3.54 IG: 2.99 ± 3.39	4.59	**0.034**	0.034
EQ-5D-5L (Mobility)	CG: 1.66 ± 0.70 IG: 1.57 ± 0.72	1.41	0.236	0.011
EQ-5D-5L (self-care)	CG: 1.47 ± 0.62 IG: 1.46 ± 0.79	0.31	0.579	0.002
EQ-5D-5L (usual activities)	CG: 1.92 ± 0.97 IG: 1.52 ± 0.70	4.93	**0.028**	**0.370** ^†^
EQ-5D-5L (pain/discomfort)	CG: 1.81 ± 0.79 IG: 1.69 ± 0.76	0.06	0.800	0.000
EQ-5D-5L (Anxiety/Dep.)	CG: 1.44 ± 0.75 IG: 1.52 ± 0.93	1.51	0.221	0.012

CG, control group; IG, intervention group; HADS, Hospital Anxiety and Depression Scale; HADS-A, Anxiety Scores; HADS-D, Depression Scores; EQ-5D-5 L, EuroQol-5 dimensions-5 levels. *Significance *p* < 0.05. ^†^Bold: effect size into the range mild–moderate (|η^2^| > 0.06).to large-high (|η^2^| > 0.14).

In terms of quality of life, a significant difference was observed only in the ‘usual activities’ subscale of the EQ-5D-5L. The IG showed greater improvement at one-month follow-up compared to the CG, and this difference was associated with a large effect size, indicating a meaningful functional benefit. No other statistically significant between-group differences were found in the remaining EQ-5D-5L subscales (i.e., mobility, self-care, pain/discomfort, and/or anxiety/depression) (see Table 4).

However, sensitivity analyses adjusting for baseline depression levels and the week of inclusion as covariates in an ANCOVA indicated that these differences were no longer statistically significant. The week of inclusion was found to have a significant effect on ‘usual activities’, suggesting that temporal variations contributed to the observed differences. These findings indicate that the initial difference between groups may partly reflect the timing of assessment rather than the intervention itself. Nevertheless, adjusting for these covariates provides a more precise estimate of the intervention effect, ensuring that observed changes are independent of baseline depression and temporal confounders. These results are in Supplementary Table S2.

## 4 Discussion

The present study aimed to examine the short-term effectiveness of a nurse-led video-based intervention in patients undergoing THA, specifically in reducing anxiety during the surgical process and improving perceived quality of life. Outcomes were compared between patients who received the video-based intervention and those who received the treatment as usual. A key finding of this study was that both groups showed similar significant improvements over time. The initially observed differences, where the IG reported fewer depressive symptoms and greater improvement in the ‘usual activities’ dimension of quality of life at one-month follow-up, were no longer statistically significant after adjusting for baseline depression. This suggests that baseline symptom severity largely explains the differences between groups, highlighting the importance of accounting for initial depression levels when assessing the impact of the intervention on functional outcomes.

Our findings indicate that patients in both the CG and the IG exhibited moderate levels of anxiety and depression, consistent with epidemiological data suggesting that up to 40% of the patients that underwent orthopedic procedures experience psychological disorders, most commonly anxiety and depression (Weinerman et al., 2023). It is important to note that our sample study did not exhibit higher levels of psychopathology at baseline. Studies consistently show that these mental health issues are not only common but also predictive of adverse surgical outcomes, including increased pain, slower physical recovery, and reduce return-to-work rates (Weinerman et al., 2023; Leo et al., 2022). Moreover, Aalders et al. (2025) concluded that anxiety and depression symptoms were independently associated with worse postoperative subjective function, pain, and quality of life after THA and longer hospital stays.

Contrary to our initial hypothesis, no statistically significant changes in anxiety or depression symptoms (as measured by the HADS) were observed between baseline and hospital discharge in either the IG or CG. We had expected that the IG would report greater improvements in emotional wellbeing at discharge compared to the CG, reflecting increased perceived safety and preparedness. However, this expectation was not supported by our findings. This result is not entirely unexpected, given the short duration of hospital stay following THA, which may be insufficient for psychological changes to manifest. These results are in line with previous literature suggesting that short hospital stays may not allow enough time for psychological improvements to manifest (Halawi et al., 2019; Oyer et al., 2021).

Additionally, no significant differences between groups were observed in the “anxiety/depression” dimension of the EQ-5D-5L, despite the improvements observed in the HADS subscales. This discrepancy could be due to the different sensitivity of both instruments to capture fine emotional changes. Additionally, it is possible that the HADS may not be sensitive enough to capture subtle improvements related to emotional security or reassurance, dimensions that were central to our intervention but may not directly translate into anxiety or depression symptom scores. Despite this, both groups did show significant improvements in overall quality of life by discharge, particularly in functional domains, although no significant differences were observed between groups at that stage. These results are in line with other studies that concluded that there were significant differences in the depression subscale (Amprachim et al., 2024).

A particularly noteworthy finding is that, while most quality-of-life improvements were comparable between groups, the initially observed difference in the ‘usual activities’ dimension of the EQ-5D-5L at one-month follow-up was no longer statistically significant after adjusting for baseline depression and week of inclusion. This indicates that the observed advantage in the IG may be largely explained by baseline depressive symptom levels rather than the intervention itself. Nevertheless, both groups showed improvements over time, and structured, video-based nursing support may still facilitate patients’ understanding, autonomy, and engagement in daily activities. This suggests that the video-based nursing-led intervention may have facilitate patients’ understanding, autonomy, and engagement in daily activities. This finding is in line with prior studies noted that nursing support is beneficial during recovery after surgery. The study of Guo et al. has noted that continuous nursing care is more effective than conventional nursing care in enhancing hip joint recovery, improving quality of life, and reducing anxiety and depression in older patients undergoing THA (Guo et al., 2022). Moreover, evidence-based nursing interventions effectively reduce postoperative complications in hip arthroplasty patients, improve limb function, alleviate neuropsychological symptoms, and significantly enhance quality of life and sleep quality. This care model offers a robust nursing approach for the postoperative management of hip arthroplasty patients (Dong et al., 2023). Our results are in line with previous studies suggesting that patients receiving the information through the video presented improvements in functionality, pain and quality of life because the video produces greater understanding and retention of information, reduction of doubts, fears and anxiety, greater autonomy and quality of life (Cetinkaya Eren et al., 2022; Kim et al., 2021; Ren et al., 2025).

These results are consistent with previous research highlighting the usefulness of audiovisual interventions as a complementary therapeutic resource in clinical settings. After adjusting for baseline depression and week of inclusion, no statistically significant differences were observed between groups, suggesting that both interventions are effective in improving quality of life, but there is no clear evidence favoring one over the other. However, video-based intervention may offer practical advantages, as it is more cost-effective and sustainable than printed materials, reducing resource use while maintaining patient understanding and engagement. The use of audiovisual media as a nursing strategy represents a low-cost, accessible and easily reproducible intervention. In addition, it can contribute to patient empowerment, promoting a better understanding of the recovery process and facilitating active participation in their care, key elements for decreasing anxiety (Cetinkaya Eren et al., 2022; Wang et al., 2023). Guided visualization, in this case supervised by nursing, can also serve as a therapeutic communication bridge, strengthening the bond between professional and patient.

### 4.1 Limitations and strengths

The current study presents some limitations that should be considered. Firstly, we only included participants undergoing elective THA for osteoarthritis, so our findings do not reflect outcomes for people undergoing THA for other reasons such as fractures. Further research is warranted to assess the applicability of this nurse-led emotional preparation intervention in urgent or fracture-related total hip replacements and across different healthcare systems. Secondly, a quasi-randomized design was used due to the logistical and structural constraints of the hospital service. While this approach may introduce some selection bias, it allowed for feasible implementation of the intervention in routine clinical practice. Finally, the follow-up period was limited to one-month post-discharge, which may not capture the long-term sustainability of improvements in anxiety, depression, or quality of life. Further studies should address long-term follow-up to ensure the stability of the changes observed.

Nevertheless, this study has several strengths. It addresses a clinically relevant and often overlooked question regarding the emotional and functional recovery of patients following THA. The intervention evaluated is pragmatic, low-cost, and delivered by nursing staff, making it potentially scalable across different clinical settings. Furthermore, the multi-timepoint assessment, including preoperative, discharge, and one-month follow-up, recaptures both early and short-term postoperative outcomes. First, the use of validated instruments (HADS and EQ-5D-5L) ensures the reliability and accuracy of outcome measurement. Second, assessments at multiple time points (pre-surgery, discharge, and one-month follow-up) allowed for evaluation of both immediate and short-term effects of the intervention. Furthermore, the study addresses clinically relevant outcomes by focusing on psychological well-being, which are often underrepresented in orthopedic postoperative care research. Finally, by examining a non-pharmacological intervention, the study contributes to the growing body of evidence supporting holistic and low-risk strategies to enhance recovery following THA.

### 4.2 Implementations for clinical practice

The findings have important clinical implications. First, they support the integration of structured psychosocial interventions into standard care for patients at risk of anxiety and depression, particularly in post-acute or rehabilitation settings. The significant and sustained reductions in psychological symptoms observed in both groups suggest that such programs can enhance recovery, improve quality of life, and potentially reduce the burden on healthcare systems by promoting faster and more complete functional recovery.

Second, after adjusting the analyses for time and baseline depression, the between-group differences were no longer statistically significant. Nevertheless, the observed trends suggest that video-based nursing interventions may still support patients’ reintegration into daily activities, which remains a key goal of rehabilitation and recovery programs. Although effectiveness was comparable to usual care, the video format offers important advantages: it is low-cost, easy to disseminate, and environmentally sustainable, as it eliminates the need for printed materials.

## 5 Conclusion

In conclusion, this video-based nursing intervention appears to be a simple and useful method for improving perceived quality of life in patients discharged after undergoing THA. Although no significant improvements were found in reducing anxiety or depression symptoms at hospital discharge in either group, both showed significant psychological improvements at the one-month follow-up. Contrary to our expectations, the IG did not show superior emotional outcomes immediately post-surgery. This may be explained by the short hospitalization period, the limitations of the HADS in detecting subtle changes in perceived emotional security, or the fact that the patients we assessed did not present significant baseline psychopathology. Nevertheless, the IG demonstrated a significantly greater improvement in the “usual activities” dimension of quality of life at one-month follow-up compared to the CG.

These findings suggest that the nursing intervention may enhance functional recovery and support patients in resuming daily activities more effectively. They also highlight the potential benefits of brief nursing-led interventions, particularly in supporting patients’ return to everyday functioning. Our findings highlight the critical importance of carefully controlling for baseline depression when evaluating interventions aimed at improving quality of life, as depressive symptom levels appear to largely explain the observed improvements, particularly in daily activities. This underscores the need for future studies to test the efficacy of targeted psychosocial interventions designed to reduce depression and, in turn, enhance functional recovery. Additionally, our results emphasize the relevance of accounting for temporal and seasonal factors, such as the week of patient inclusion, which may influence prognosis and contribute to variability in outcomes. Careful consideration of these covariates is essential to accurately assess the true effects of interventions and to optimize postoperative care strategies for hip arthroplasty patients.

Further studies should explore the long-term outcomes and optimize the implementation of such interventions in clinical practice. Also, use of more sensitive or qualitative measures to better assess perceived safety, emotional readiness, and patient experience.

## Data Availability

The raw data supporting the conclusions of this article will be made available by the authors, without undue reservation.
