# Supplementary material for: The impact of nursing-led emotional preparation on mental health after total hip arthroplasty
Source: Front Psychol. 2025 Nov 5;16:1693111. doi: 10.3389/fpsyg.2025.1693111 (PMC12626945; doi:10.3389/fpsyg.2025.1693111)
Supplement: Supplementary file 1 [file Table_1.DOCX]

**Supplementary material**

**Table S1**

Estimated marginal means of HADS-A and HADS-D at discharge and 1-month follow-up, adjusted for baseline depression and week of inclusion.

|  | Group | n | Adjusted Mean (EMM) | std. Error | 95% CI | | Difference inter-group | 95% CI | | F | *p* | Partial η² |
| --- | --- | --- | --- | --- | --- | --- | --- | --- | --- | --- | --- | --- |
|  | | | | | Lower Bound | Upper Bound |  | Lower Bound | Upper Bound |  | | |
| HADS |  | | | | | | | | | | | |
| HADS-A post | CG | 64 | 7.14 | 0.46 | 6.24 | 8.04 | 0.27 | -1.01 | 0.77 | .174 | .677 | .001 |
|  | IG | 67 | 6.87 | 0.45 | 5.99 | 7.75 |  |  |  |  |  |  |
| HADS-A 1m | CG | 64 | 3.98 | 0.41 | 3.18 | 4.78 | 0.48 | -0.63 | 1.63 | .700 | .404 | .006 |
|  | IG | 67 | 3.50 | 0.40 | 2.71 | 4.28 |  |  |  |  |  |  |
| HADS-D post | CG | 64 | 5.72 | 0.38 | 4.95 | 6.48 | 0.32 | -1.40 | 0.77 | .338 | .562 | .003 |
|  | IG | 67 | 6.03 | 0.37 | 5.29 | 6.78 |  |  |  |  |  |  |
| HADS-D1m | CG | 64 | 3.90 | 0.39 | 3.11 | 4.68 | -0.65 | -1.76 | 0.46 | 1.327 | .252 | .010 |
|  | IG | 67 | 3.25 | 0.38 | 2.48 | 4.02 |  |  |  |  |  |  |

Note: CI = Confidence Interval; p-values are Bonferroni-adjusted. CG =control group. IG= intervention group. HADS= Hospital Anxiety and Depression Scale. HADS-A= Anxiety Scores. HADS-D= Depression Scores.EMM= Adjusted Mean

**Table S2.** ANCOVA results for EQ-5D-5L subscales: effects of baseline depression and week of inclusion between groups (IG vs CG) at post and at 1 month follow-up.

| **Subscale** | **Timepoint** | **Covariate / Effect** | **F** | **p** | **Partial η²** |
| --- | --- | --- | --- | --- | --- |
| Mobility | Post | Baseline depression | 1.164 | 0.283 | 0.009 |
|  |  | WEEK | 22.801 | **<0.001** | 0.153 |
|  |  | GROUP | 0.011 | 0.916 | 0.000 |
| Mobility | 1 month | Baseline depression | 2.589 | 0.110 | 0.020 |
|  |  | WEEK | 20.648 | **<0.001** | 0.141 |
|  |  | GROUP | 0.027 | 0.871 | 0.000 |
| Self-care | Post | Baseline depression | 2.823 | 0.095 | 0.022 |
|  |  | WEEK | 1.465 | 0.228 | 0.011 |
|  |  | GROUP | 0.008 | 0.929 | 0.000 |
| Self-care | 1 month | Baseline depression | 4.767 | **0.031** | 0.036 |
|  |  | WEEK | 0.253 | 0.616 | 0.002 |
|  |  | GROUP | 0.088 | 0.767 | 0.001 |
| Usual activities | Post | Baseline depression | 5.477 | **0.021** | 0.042 |
|  |  | WEEK | 8.879 | **0.003** | 0.066 |
|  |  | GROUP | 0.519 | 0.473 | 0.004 |
| Usual activities | 1 month | Baseline depression | 7.858 | **0.006** | 0.059 |
|  |  | WEEK | 8.094 | **0.005** | 0.060 |
|  |  | GROUP | 3.021 | 0.085 | 0.023 |
| Pain/discomfort | Post | Baseline depression | 0.650 | 0.422 | 0.005 |
|  |  | WEEK | 1.866 | 0.174 | 0.015 |
|  |  | GROUP | 0.066 | 0.797 | 0.001 |
| Pain/discomfort | 1 month | Baseline depression | 1.878 | 0.173 | 0.015 |
|  |  | WEEK | 0.860 | 0.355 | 0.007 |
|  |  | GROUP | 0.070 | 0.792 | 0.001 |
| Anxiety/depression | Post | Baseline depression | 4.286 | **0.040** | 0.033 |
|  |  | WEEK | 0.396 | 0.531 | 0.003 |
|  |  | GROUP | 0.024 | 0.876 | 0.000 |
| Anxiety/depression | 1 month | Baseline depression | 2.473 | 0.118 | 0.019 |
|  |  | WEEK | 0.003 | 0.954 | 0.000 |
|  |  | GROUP | 1.406 | 0.238 | 0.011 |

Note: GROUP: Intervention group (IG) vs Control group (CG). WEEK: Covariate representing week of participant inclusion. Partial η²: Reported effect size; values interpreted as small (~0.01), medium (~0.06), or large (~0.14) according to Cohen’s conventions. Adjusted means are estimated marginal means controlling for baseline depression and week of inclusion (WEEK).p-values are Bonferroni-adjusted for multiple comparisons.
